# Enhanced Microwave Trapping and Loss Capabilities of TiN/RGO/PDMS Metacomposites across a Wide Range of Temperatures

**DOI:** 10.34133/research.0972

**Published:** 2025-10-29

**Authors:** Haoxu Si, Shuai Zhang, Yaqin Ding, Chongyang Chai, Shuaishuai Zhou, Cuiping Li, Jingwei Zhang, Chunhong Gong

**Affiliations:** ^1^ National and Local Joint Engineering Research Center for Applied Technology of Hybrid Nanomaterials, Henan University, Kaifeng 475004, China.; ^2^ Institute of Functional Polymer Composites, College of Chemistry and Molecular Sciences, Henan University, Kaifeng 475004, China.; ^3^ School of Energy Science and Technology, Henan University, Kaifeng 475004, China.

## Abstract

According to the relationship between unit size and wavelength, electromagnetic waves (EMWs) in metacomposites can be effectively absorbed through inherent loss mechanisms. However, limited loss ability has emerged as a primary obstacle to achieving efficient EMW absorption (EMWA) performance. The challenge of enhancing the attenuation of corresponding metacomposites remains important. In this study, we prepared elaborate titanium nitride/reduced graphene oxide units with controllable conductivities and sizes, which formed the microwave antireflective surface of metacomposites. This configuration contributes to improved impedance matching and reduced temperature sensitivity concerning EMWA performance. More importantly, EMWs experience repeated collisions and loss, along with multiple reflections and scattering within metacomposites. This phenomenon extends the transmission pathways of EMWs, thereby increasing additional loss capacity. Consequently, these metacomposites exhibited exceptional EMWA performance across a broad temperature range (298 to 573 K), while maintaining a low filler content of 1.0 wt% units. Henceforth, our findings not only provide an advantageous pathway for high design flexibility in EMWA but also pave the way for new research directions and possibilities regarding advanced lightweight metacomposites that optimize EMWA over an extensive temperature range.

## Introduction

With the explosive development of aerospace vehicles and electronic devices, the undesirable electromagnetic (EM) pollution and radiation, as well as the considerable heat emission, will severely reduce their service life and functionality and even threaten human health [1–4]. Therefore, it is of great significance to develop a new type of absorbents with an effective and stable EM wave absorption (EMWA) performance in a wide temperature range. According to the Debye equation: $\varepsilon^{\prime \prime }=\varepsilon_{p}^{\prime \prime }+\varepsilon_{c}^{\prime \prime }=\left(\varepsilon_{s}-\varepsilon_{\infty }\right)\frac{\omega \tau }{1+\omega^{2}\tau {\left(T\right)}^{2}}+\frac{\sigma \left(T\right)}{\varepsilon_{0}\omega }$, where $\varepsilon_{s}$, $\varepsilon_{0}$, $\varepsilon_{\infty }$, τ, ω, and σ are the static permittivity, permittivity of free space, relative permittivity at the high-frequency limit, relaxation time, angular frequency, and conductivity [$\sigma \left(T\right)=Ae^{-E/2\mathrm{kT}}$], respectively, the dielectric loss predominantly stems from the polarization loss ($\varepsilon_{p}^{\prime \prime }$) and conductive loss ($\varepsilon_{c}^{\prime \prime }$), both of which are temperature functions [5]. The conductive loss shows the strong temperature dependence, attributed to the fact that the electrons are thermally activated to hop and migrate, and boost conductivity at high temperatures, yet causing impedance mismatching [6]. Inversely, the polarization loss is decreased, compensating for the increase of conductive loss, whittling the increase of permittivity, and further contributing to impedance matching at high temperatures [7–9]. Previous works mainly focused on the adjustment of micro-scale structures and components, and optimized impedance matching and decreased temperature sensitivity, resulting in a weak loss ability [7–12]. For example, Yin et al. designed a SiC fibers (SiC_f_)/silicon nitride (Si_3_N_4_) composite, a sandwich-like reduced graphene oxide (RGO)/Si_3_N_4_ composite, and mesoporous carbon hollow microspheres with red blood cell-like morphology, and the EMWA properties showed weaker temperature dependence due to the low content of conductive phase in the composite [7–9]. However, it also gave rise to the weak loss ability in a wide temperature range. Thus, how to break through the temperature dependence of EMWA properties and optimize the loss capacity in a wide temperature range is still a tough challenge.

Recently, to address the issue, the conversion of conventional conductive networks into discrete absorbing units creates a novel method to increase loss ability and optimal impedance matching via the formation of local conductive networks in the composites. For example, different from the traditional way of grinding into powder and filling evenly, Gong et al. constructed subwavelength-scale functional units with various micro- and mesoscopic structures, such as flexible titanium nitride (TiN) nanofiber membranes (TNM_s_), graphene nanosheet cluster, reduced graphene oxide aerogel powders (GAP), RGO@carbon spheres, and ATO/SiO_2_ spheres, and the corresponding metacomposites contributed to optimizing the impedance matching characteristics and local loss capabilities, as well as EMWA performance [13–17]. Besides, the discrete functional units provide anti-reflective characteristics to EMWs, which are attributed to the destruction of the macroscopic conductive network, and free electrons are unable to hop between the functional units, leading to temperature-insensitive EMWA performances at high temperatures [16]. Due to the destruction of a macroscopic conductive network of metacomposites, which transforms into mesoscale functional units, free electrons can simultaneously hop and migrate inside functional units; thus, it has both optimal impedance matching and strong loss ability, overcoming the traditional material limitations [16].

Different from microscale materials or macroscale metamaterials, metacomposites uniquely leverage both the inherent EM parameters of their constituents and the properties of artificial structures to optimize EMWA performance. By integrating the advantages of traditional materials with those of metamaterials, metacomposites achieve tunable EM properties and exceptional design flexibility, thereby addressing the fundamental limitations associated with conventional material design [18,19]. Essentially, the tunability of EM properties in metacomposites arises not only from material composition but also from the intentional macro arrangement and microstructural design of functional units. Furthermore, the discrete distribution of functional units can attenuate intrinsic EM parameters, thereby reducing temperature sensitivity and improving the EMWA performance across a wide temperature range [20]. From the standpoint of the interplay between matter and electromagnetic waves (EMWs), though subwavelength-scale functional units produced a good anti-reflection effect on EMWs, an escalation in the scale of functional units engenders a plethora of reflections and scattering phenomena concerning the incident EMWs [17,21,22]. Whether this intricate interaction enhances or weakens their capacity for energy dissipation depends on the competition between the impedance matching and attenuation capabilities. In other words, the functional unit provides good impedance matching characteristics; the additional multiple reflections and scattering between the functional units will be generated based on the conductivity and size of the functional unit, enhancing the attenuation capability and EMWA intensity [23,24]. However, when the size or conductivity of functional units surpasses a certain threshold, the internal reflections and scattering will become surface reflection, resulting in diminishing the attenuation capabilities. Meanwhile, when the dimensions of functional units are obviously smaller than the operational wavelength, metacomposites can be considered as an equivalent homogeneous medium. In this scenario, they exhibit a limited capacity for internal reflection and scattering, which ultimately leads to suboptimal loss characteristics [24–26]. As of now, the investigation into subwavelength-scale metacomposites is still in its preliminary stages, and the relationship between functional unit characteristics and loss capacity has yet to be elucidated.

TiN has emerged as a promising high-temperature absorbing material due to its compelling combination of low density, exceptional temperature resistance (*T*_m_ = 3,223 K), corrosion resistance, and good electrical conductivity [13]. Furthermore, TiN nanofibers (NFs) create abundant heterointerfaces, which induce multipolarization effects, markedly enhancing interfacial polarization and the overall attenuation of EMWs [5]. Herein, TiN NFs/RGO units with tailored conductivities and sizes were fabricated, and constructed the metacomposites with high design freedom. This strategy overcomes the limitations in tunability associated with conventional metamaterials, enhancing the dissipation of EM energy while maintaining excellent impedance matching. Crucially, the precise configuration of discretely distributed units facilitates multiple reflections and scattering of EMWs, resulting in repeated energy collisions. This process extends the propagation paths of EMWs and increases interaction opportunities with functional units, thereby introducing additional attenuation mechanisms that substantially improve microwave-trapping capabilities within the metacomposites. Consequently, due to their exceptional anti-reflective properties and multimechanistic dissipation capabilities, the as-fabricated metacomposites demonstrate outstanding EMWA performance across a broad temperature range (298 to 573 K), even at a low unit filler loading of 1.0 wt%. These findings not only provide an in-depth understanding of the interplay among functional units, loss mechanisms, and EMWA performances but also pave the way for designing advanced metacomposites that are lightweight and efficient for EMWA across varying temperatures.

## Results and Discussion

### Morphology and composition

**Fig. 1.**
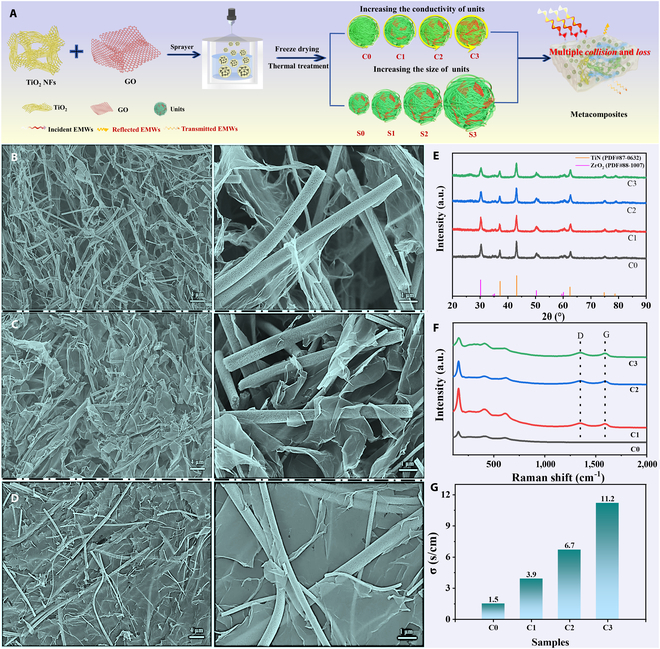
Preparation process of metacomposites (A). SEM images of C1 to C3 (B to D): (B) C1, (C) C2, (D) C3; XRD (E), Raman spectrum (F), and conductivity (G) of C0 to C3.

The TiN/RGO/PDMS metacomposites were fabricated through electrostatic spinning, electrostatic spraying, and nitridation pyrolysis, as illustrated in Fig. 1A. The mixed solution of TiO_2_ NFs and graphene oxide (GO) dispersion with different mass ratios were prepared by high-speed shear emulsifier and magnetic stirring. Subsequently, the TiN/RGO units were obtained via electrostatic spraying and nitridation pyrolysis. Herein, to obtain additional loss mechanisms (repeated collisions and loss, multiple reflections and scattering, etc.), units with different conductivities and sizes were constructed, thereby achieving better EMWA properties over a wider temperature range, overcoming the core issue of mutual restraint between good impedance matching and strong loss capacity.

The morphology and components of C0 to C3 are ascertained in Fig. 1B to D. The morphology of C0 is preliminarily explored by a scanning electron microscope (SEM) (Fig. S1); it reveals the well-preserved NFs, which is consistent with previous work [13]. Other C1 to C3 samples show that TiN NFs and lamellar RGO interlock and interweave with each other, further forming a large number of network structures. With the increase of RGO ratio, the lamellar RGO inside the units from C1 to C3 increases substantially​, and the lamellar RGO almost completely wraps TiN NFs, which improves the density of the network structure. From a microscopic point of view, one-dimensional (1D) NFs are bonded to each other to form a preliminary network structure, and the NFs and 2-dimensional (2D) lamellar RGO are cross-linked to form 3-dimensional (3D) conductive networks inside the units, which is beneficial for migrating and hopping of charges, improving the conductive loss ability.

To analyze the phase composition, x-ray diffractometry (XRD) and Raman tests are performed. In Fig. 1E, the diffraction peaks display both high crystallinity and purity. Obviously, the diffraction peaks at 2*θ* = 37.0°, 43.0°, 62.5°, 74.9°, and 78.8° correspond to the (111), (200), (220), (311), and (222) crystal faces of the cubic phase TiN (JCPDS No. 87-0632), and 2*θ* = 30.2°, 34.8°, 35.2°, 50.4°, 59.6°, and 60.2° correspond to (101), (002), (110), (112), (103), and (211) crystal faces of the quadrangular phase ZrO_2_ (JCPDS No. 88-1007), respectively. Meanwhile, the characteristic peaks in Fig. 1F at 153, 248, 412, and 607 cm^−1^ are attributed to TiN, indicating the formation of TiN, in agreement with the XRD characterization results. Besides, 2 distinctive peaks (1,344 and 1,594 cm^−1^) belong to the D- and G-band of RGO, confirming the reduction of GO [27,28]. In addition, the conductivity gradually increases (C0: 1.5 S cm^−1^, C1: 3.9 S cm^−1^, C2: 6.7 S cm^−1^, and C3: 11.2 S cm^−1^) with the addition of the RGO ratio (Fig. 1J). The increased conductivity is mainly attributed to 2 factors: (a) RGO has a positive effect on the improvement of overall conductivity, and (2) the 3D network structures formed by the interweaving of more RGO and TiN NFs will be more abundant and tighter inside the units.

### Influence of conductivity of different units on the EMWA properties

**Fig. 2.**
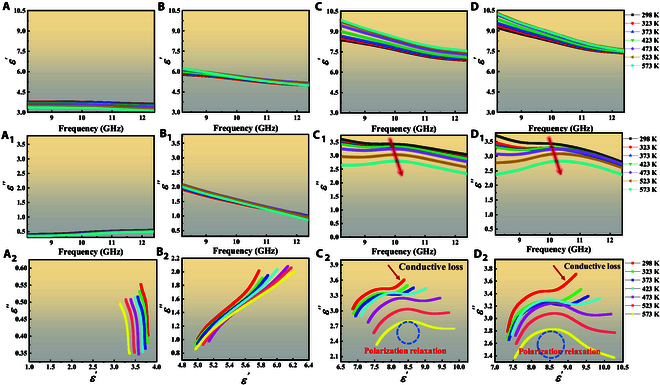
Real part (A to D), imaginary part (A_1_ to D_1_), and Cole–Cole curves (A_2_ to D_2_) of Cx/PDMS metacomposites at 298 to 573 K: (A to A_2_) C0, (B to B_2_) C1, (C to C_2_) C2, and (D to D_2_) C3.

The influence of conductivity on the EM parameters is investigated thoroughly by complex permittivity and permeability, which plays a decisive role in the EMWA properties [29]. Thus, owing to the nonmagnetic component of Cx/PDMS metacomposites, the function of complex permeability can be negligible [30]. The *ε*′ values of Cx/PDMS metacomposites (Fig. 2A to D) present a gradual decrease trend with the varied frequency, indicating a representative frequency dispersion behavior [31]. This is mainly due to the fact that the dipole steering or vibration gradually fails to keep up with the change in frequency, which leads to the weakening of polarization ability. Simultaneously, the *ε*′ and *ε″* values increase with the increase of RGO (Fig. 2A to D_1_), as displayed in Fig. S2, which are mainly ascribed to the high dielectric permittivity of RGO. The more lamellar RGO can combine with TiN NFs to form the abundant 3D conductive network inside units, further resulting in the increase of conductivity and conductive loss [31–33]. Nevertheless, the C2/PDMS and C3/PDMS metacomposites emerge the atypically resonance peaks within the high-frequency region, which are more apparent in tan𝛿ε (Fig. S3). These features signify the activation and strengthening of multiple polarization relaxation mechanisms, specifically interfacial polarization and dipole polarization [32,34]. The underlying origin of this behavior can be attributed to the continuous increase of RGO content; 2D RGO nanosheets and 1D TiN NFs become intimately intertwined and overlapping, constructing the interconnected architecture. This multidimensional arrangement introduces abundant active sites and highly heterogeneous interfaces, simultaneously establishing extensive conductive pathways. Consequently, these microstructural characteristics promote the intense interfacial polarization and dipole polarization.

Furthermore, the EM dissipation behavior can be interpreted through the Debye relaxation theory. According to the Cole–Cole curves $\begin{array}{c} \left({\left(\varepsilon^{\prime }-\frac{\varepsilon_{s}+\varepsilon_{\infty }}{2}\right)}^{2}+{\left(\varepsilon^{\prime \prime }\right)}^{2}={\left(\frac{\varepsilon_{s}-\varepsilon_{\infty }}{2}\right)}^{2}\right) \end{array}$, each semicircular arc in the *εʹ–ε″* plot corresponds to a polarization relaxation process [35]. As depicted in Fig. 2C_2_ and D_2_, the presence of multiple distorted semicircles accompanied by linear tails signifies the coexistence of diverse polarization mechanisms (such as interfacial and dipole polarization) and conductive losses. It forms a sharp contrast with the curves in Fig. 2A_2_ and B_2_, where the curves of C0/PDMS and C1/PDMS metacomposites are almost straight lines attributed to the low content RGO addition, further confirming the enhanced polarization relaxation within C2/PDMS and C3/PDMS metacomposites [10,34,35]. By increasing the RGO addition, the local conductive networks of units are formed. The adjacent charges at the interfaces interact with each other, enhancing the local electric field intensity and resulting in a more pronounced interfacial polarization phenomenon, in agreement with the abovementioned analysis [36–38]. Besides, the effect of temperature on polarization loss involves competing mechanisms. On the one hand, higher temperatures generally promote interfacial charge accumulation, potentially enhancing polarization loss. Conversely, thermal activation facilitates the orientational rotation of dipoles, which alleviates polarization lag and reduces the associated frictional resistance [3,9,39]. This is further aided by the external thermal energy, which lowers the activation barrier required to overcome orientational resistance [39]. Although increased thermal motion can also introduce a disordering effect that impedes dipole alignment, the result is often a decrease in the energy required to overcome internal friction. According to the abovementioned analysis, the semicircular arc of Cole–Cole curves can increase with the boosted temperature, as shown in Fig. 2C_2_ and D_2_.

Intriguingly, the Cx/PDMS metacomposites demonstrate different temperature dependences with increasing RGO contents. The *ε″* values of C0/PDMS and C1/PDMS metacomposites basically remain the same with the changing temperature, which is due to the low RGO content and poor polarization loss caused by the less heterogeneous interfaces. Inversely, the *ε″* values of C2/PDMS and C3/PDMS metacomposites show observable changes with the varying temperatures, which decrease as the temperature rises. It is ascribed to the increased interfacial and dipole polarization produced by the added heterogeneous interfaces (RGO and TiN NFs) and functional groups at a high RGO content, which suppresses the positive effect of conductive loss on temperature in the units from a microscopic perspective. With the rise of temperature, more electrons are thermally activated to hop and migrate across potential barriers, further increasing conductivity and conductive loss [39]. Yet, the higher conductivity will generate the local loss, presenting a negative role to impedance matching and reflecting the incident EMWs on the surface of Cx/PDMS metacomposites [16]. Inversely, the additional multiple effective reflections and scattering will occur between the units, increasing the consumption of EMWs, turning a disadvantage into an advantage.

**Fig. 3.**
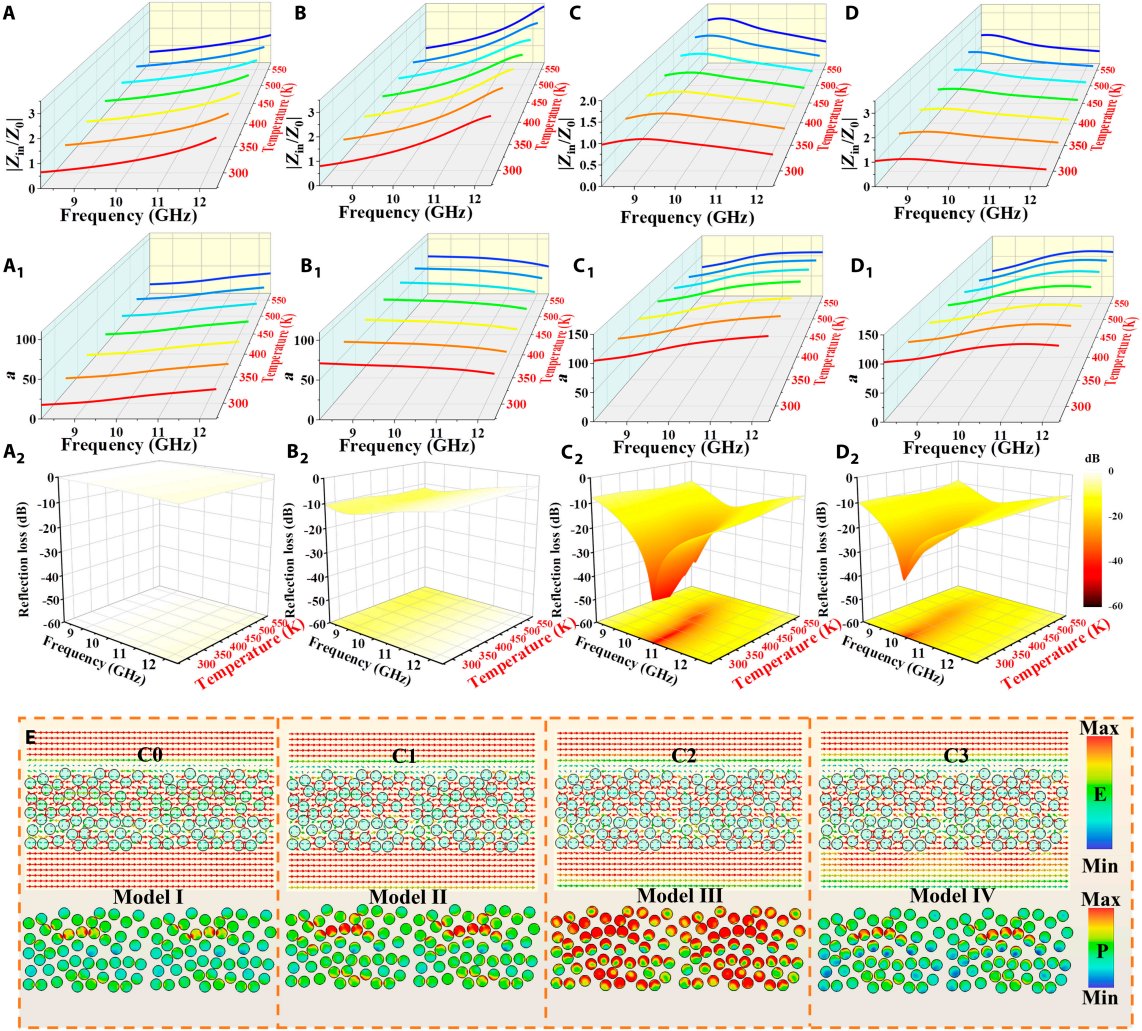
|*Z*_in_/*Z*_0_| values (A to D), attenuation constant (A_1_ to D_1_), and reflection losses (A_2_ to D_2_) of Cx/PDMS metacomposites at 298 to 573 K with 2.7 mm: (A to A_2_) C0, (B to B_2_) C1, (C to C_2_) C2, and (D to D_2_) C3, the electric field and power loss density power loss density of Cx/PDMS metacomposites models at 10.0 GHz (E). The arrows correspond to the vector of electric field.

Excluding the abovementioned intrinsic loss capacity of units, the multiple reflections and scattering between units boost the transmission path of EMWs and essentially increase the interaction between EMWs and units. With the incremental conductivity from C0 to C2, the repeated collisions of EMWs between units will increase, which primarily affects the dissipation pathways of EM energy. These collisions can scatter and redistribute EM energy, potentially enhancing its conversion into thermal energy or other forms of energy, further contributing to the improvement of the overall loss ability and EMWA performance. Continuing to boost the conductivity (C3), the surface reflections are generated, damaging the EMWA performances ascribed to impedance mismatching, as attested in Fig. 3A to D. The comparison of impedance matching (*Z* = |*Z*_in_/*Z*_0_|) shows that C2/PDMS metacomposites (Fig. 3C) gradually approach the ideal value of 1 [40]. This superior impedance matching minimizes surface reflection, permitting more incident EMWs to enter the interior of C2/PDMS metacomposites as much as possible [41,42]. The penetrating EMWs are then effectively attenuated by ample loss mechanisms, such as multiple reflections and scattering, conductive loss, and polarization loss, thereby enhancing EMWA [32,43–45]. The loss capacity of samples can be evaluated by attenuation constant $\begin{array}{c} \begin{array}{c} \begin{array}{c} \left(\alpha =\frac{\sqrt{2}\pi f}{c}\times \sqrt{\left(\mu^{\prime \prime }\varepsilon^{\prime \prime }-\mu^{\prime }\varepsilon^{\prime }\right)+\sqrt{{\left(\mu^{\prime \prime }\varepsilon^{\prime \prime }-\mu^{\prime }\varepsilon^{\prime }\right)}^{2}+{\left(\mu^{\prime }\varepsilon^{\prime \prime }+\mu^{\prime \prime }\varepsilon^{\prime }\right)}^{2}}}\right) \end{array} \end{array} \end{array}$, as shown in Fig. 3A_1_ to D_1_ [46,47]. A higher α corresponds to a greater capacity for EMW attenuation. When the conductivity of units continues to increase, although the attenuation capacity of C3/PDMS metacomposite (Fig. 3D_1_) has increased, the impedance matching characteristics of C3/PDMS metacomposite (Fig. 3D) begin to deviate. It is mainly because the skin effect caused by excessively high conductivity will become more​​strong​​, resulting in the deterioration of impedance matching characteristics and the reflection of incident EMWs, as well as further poor EMWA performances [39,48].

As substantiated in Fig. 3A_2_ to D_2_, the C2/PDMS metacomposites have an excellent EMWA performance (−44.1 dB) at a relatively low filling ratio (1 wt%), and the effective absorption bandwidth (EAB) can almost cover the entire X-band, compared with those of other Cx/PDMS metacomposites (C0: −2.45 dB; C1: −6.54 dB; C3: −36.9 dB). The inferior EMWA performances of C0/PDMS metacomposites and C1/PDMS metacomposites are due to the insufficient overall loss capacity caused by the weak conductivity and fewer polarization sites, corresponding to the low dielectric constant. With the more RGO content of C3 units, the density of the conductive network further increases, and the EMWA performances begin to decrease. When the conductivity exceeds a certain level, the skin effect will occur on the surface to reflect EMWs, resulting in the weakening of impedance matching characteristics and an increase of ineffective reflection; thus, the overall EMWA performance shows a trend of attenuation, as shown in Fig. 3E. Models I and II show the low electric field intensity and power loss density, attributed to the inferior conductivity of C0/C1. Model III emerges remarkably changed electric field intensity, power loss distribution, and mutual effect around the C2, indicating the strong interaction between EMWs and C2 units, further increasing the loss of EM energy. With the excessively increased conductivity, Model IV exhibits weak electric field strength, power loss density, and interaction. Thus, the metacomposites with different conductivities mainly influence the repeated collision, multiple reflections and scattering of EMWs between units, and the microwave-trapping ability.

Furthermore, the EMWA performances of C2/PDMS metacomposites exhibit gradual degradation as temperature increases. This behavior can be primarily attributed to the isolated nature of the conductive units, which form a discontinuous conductive network throughout the polymer matrix. The polarization lag associated with the orientation rotation of dipoles induced by thermal activation is alleviated as temperature increases, resulting in a reduction of frictional resistance [3,9,39,49]. Concurrently, the disordering effect caused by the thermal motion of dipoles improves with elevated temperatures, which impedes orientation rotation [39]. Furthermore, the external environment supplies additional energy to polarons, thereby decreasing the energy required to overcome orientational resistance. Consequently, less energy is needed to surmount internal friction as temperature rises, and this leads to a reduction in polarization loss. Notably, even at an elevated temperature of 573 K, C2/PDMS metacomposites retain an exceptional EMWA of up to 99%. This outstanding EMWA performance is achieved through synergistic loss mechanisms including repeated collisions, multiple reflections and scattering, conductive loss, and polarization loss. The multiple reflection and scattering effects prolong the propagation path of EMWs within the metacomposite, enhancing energy interaction between EMWs and functional units. This process effectively traps incident EMWs, facilitating progressive attenuation until the energy is fully consumed. Moreover, the conductive loss contributes through Joule heating induced by carrier migration, and polarization loss (interfacial and dipole polarization) remains active even at high temperatures. Together, these mechanisms synergistically enhance the microwave-trapping capacity, ensuring the robust EMWA performance under high-temperature conditions.

### Influence of size of different units on EMWA properties

**Fig. 4.**
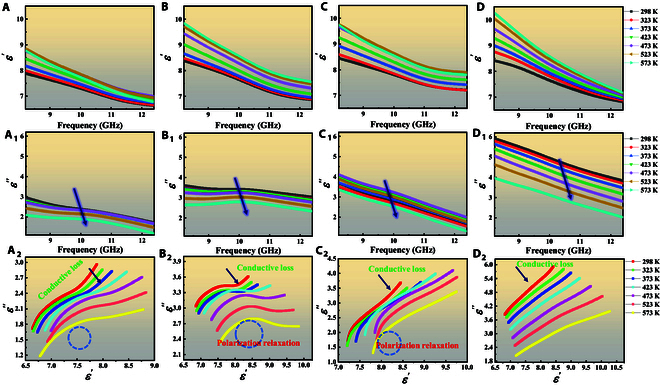
Real part (A to D), imaginary part (A_1_ to D_1_), and Cole–Cole curves (A_2_ to D_2_): (A to A_2_) S0, (B to B_2_) S1, (C to C_2_) S2, and (D to D_2_) S3.

To explore the effects of additional loss mechanisms on dielectric properties, units with different scales are designed (Figs. S4 and S5), which contain the same components, ensuring the coincident intrinsic properties. The Sx/PDMS metacomposites exhibit temperature–frequency response characteristics, as illustrated in Fig. 4. With the increase of unit size from S0/PDMS to S3/PDMS metacomposites, both *ε*′ and *ε″* values tend to increase (Fig. 4A to D_1_ and Fig. S6), indicating the ever-rising dielectric loss, which is consistent with tan 𝛿e values (Fig. S7) [10]. The tan 𝛿e value gradually increases from ~0.21 to 0.37 of S0/PDMS metacomposites to ~0.40 to 0.70 of S3/PDMS metacomposites. It is mainly due to the greater loss ability and enhanced multiple effective reflections/scattering between the units with the increase of size. Concretely, on the one hand, the conductive channels for free electron hopping and migrating are boosted with the increase in unit size, which is conducive to the improvement of conductive loss ability [3]. The movement and aggregation of free electrons at the heterogeneous interfaces between TiN NFs and RGO are also beneficial for the enhancement of the polarization effect [50]. On the other hand, the longer 3D transmission paths can provide more sites to reflect/scatter/collide EMWs between the units with the increase in size, further increasing the collision that dissipates energy and enhances microwave trapping [49].

The mechanism of dielectric loss is further elucidated by Cole–Cole plots (Fig. 4A_2_ to D_2_), in which distinct semicircular arcs accompanied by slender linear tails are observed, suggesting the simultaneous presence of polarization loss and conductive loss [51]. Specifically, the S0/PDMS and S1/PDMS metacomposites exhibit well-defined semicircular curves, indicating a pronounced polarization loss. In contrast, the Cole–Cole plots of S2/PDMS and S3/PDMS metacomposites display gradual attenuation of semicircular features along with pronounced extension of the linear tail. This morphological evolution implies a shift in the dominant loss mechanism; the number and mobility of internal charge carriers are enhanced as the unit size increases, promoting the formation of conductive networks within the units, contributing to the enhancement of conductive loss.

In addition, the dielectric properties of Sx/PDMS metacomposites show a decreasing trend with the rise of temperature, and this phenomenon becomes more obvious as the scale of units increases. This is mainly due to the gradual increase in conductivity caused by more thermally activated electrons, but the rich 3D conductive networks are confined to the interior of units, which constitutes an interrupted macroscopic conductive path; thus, the macroscopic conductive loss is weak [16]. Meanwhile, owing to the decrease of energy expended by dipoles in rotation or orientation at high temperatures, the polarization loss decreases; thus, the above trend of change is displayed [39].

**Fig. 5.**
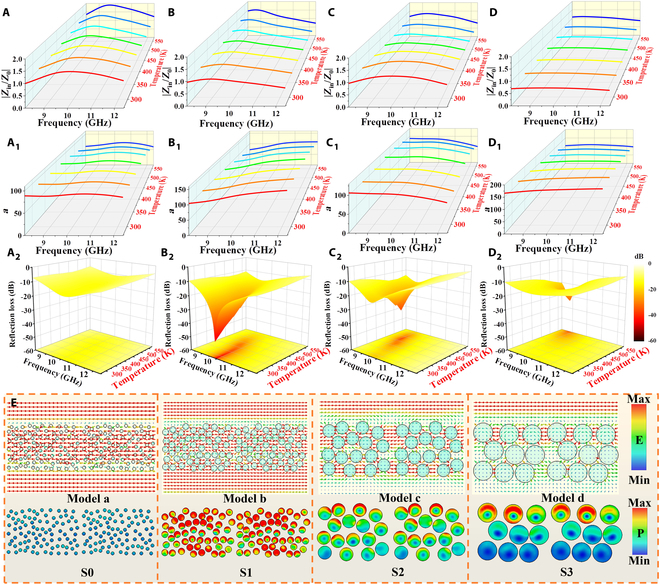
|*Z*_in_/*Z*_0_| values (A to D), attenuation constant (A_1_ to D_1_), and reflection losses (A_2_ to D_2_) of Sx/PDMS metacomposites at 298 to 573 K with 2.8 mm: (A to A_2_) S0, (B to B_2_) S1, (C to C_2_) S2, and (D to D_2_) S3, the electric field and power loss density power loss density of Sx/PDMS metacomposites models at 10.0 GHz (E). The arrows correspond to the vector of electric field.

*Z* and α play vital roles in evaluating the EMWA performance of metacomposites, as displayed in Fig. 5A to D and Fig. 5A_1_ to D_1_ [30,52,53]. The *Z* values of S0/PDMS metacomposites (Fig. 5A, 1.2 to 1.7) gradually deviate from the optimal range (0.8 to 1.2) at room temperature [17]. By increasing the unit size, the *Z* values of S1/PDMS metacomposites (Fig. 5B) improve and tend to be closer to the ideal value of 1. Yet, with a further unit size increase in S2/PDMS and S3/PDMS metacomposites, their *Z* values (Fig. 5C and D) become worse, indicating the surface reflection of incident EMWs, which is attributed to the enhanced local eddy current caused by longer electron transport paths [49]. Simultaneously, Fig. 5A_1_ to D_1_ show that the loss ability of Sx/PDMS metacomposites gradually becomes stronger as the unit size increases, which can be mainly ascribed to the conductive loss and more collisions accompanied by multiple reflections and scattering, extending the paths of EMW attenuation. Owing to its optimal *Z* ≈ 1 and substantial *α*, the S1/PDMS metacomposite is predicted to exhibit exceptional EMWA performances.

Besides, the *Z* values of S0/PDMS and S1/PDMS metacomposites (Fig. 5A and B) gradually deviate from the appropriate range (0.8 to 1.2) with the increased temperature (298 to 573 K). Differently, the *Z* values of S2/PDMS and S3/PDMS metacomposites (Fig. 5C and D) remain close to 1 with the varied temperature, which is ascribed to the comprehensive effect of the increased local loss and decreased polarization loss. Fig. 5A_2_ to D_2_ show the EMWA performances of Sx/PDMS metacomposites at 2.8 mm in the 298 to 573 K range. At 298 K, the corresponding reflection loss value demonstrates a positive correlation with *Z* values, such as S0: −15.5 dB, S1: −48.1 dB, S2: −21.2 dB, and S3: -14.5 dB. Simultaneously, the EAB of S1/PDMS metacomposites basically covers the entire X-band. However, both S2/PDMS and S3/PDMS metacomposites exhibit marked enhancement in EMWA performances with increasing temperature. For instance, the reflection loss of S3/PDMS metacomposites progressively improves from −15.0 dB at 323 K to −33.8 dB at 573 K, demonstrating a strong positive temperature dependence. This remarkable improvement stems from a multiscale synergistic loss mechanism. At the microscopic scale, intrinsic conductive loss and polarization loss contribute collectively to energy dissipation. More importantly, from a mesoscopic perspective, repeated collisions accompanied by multiple reflections and scattering plays a decisive role in achieving superior EMWA [54]. As the temperature rises, the conductivity of units increases, promoting the formation of more efficient reflective and scattering sites within metacomposites [16]. This expanded network prolongs the propagation path of incident EMWs, creating enhanced transmission channels and intensifying the interaction between EMWs and units. Consequently, the loss capacity is substantially augmented, leading to the outstanding enhancement in EMWA performance with the varied temperature.

Besides, when the unit size is small, the reflections and scattering of EMWs between units will become weak, which is not conducive to the consumption of EMWs. With the increase of unit size, the transmission paths of EMWs are increased, enhancing the multiple reflections and scattering of EMWs between units, contributing to the enhancement of interaction between units and EMWs and attenuating more EM energy. Besides, the active sites caused by the collision surface will provide more opportunities for EMWs to enter the interior of material and then be attenuated through local loss. However, when the size is too large, the surface reflection of S2/PDMS or S3/PDMS metacomposites is increased. Meanwhile, the above results can be also confirmed by electric field vector distributions; Fig. 5E (S1/PDMS metacomposites: Model b) directly demonstrates a marked increase in electric field intensity, power loss distribution, and mutual effect, compared with other metacomposites, implying the stronger loss ability both between and within units. Thus, the multiple reflections and scattering between units attenuate more EMWs until they are completely consumed, achieving an excellent EMWA performance in a wider temperature range.

**Fig. 6.**
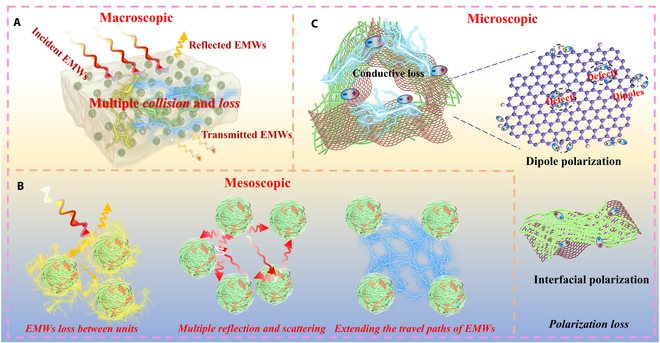
Schematic illustration of the EMWA mechanism of metacomposites from a multiscale perspective: (A) macroscopic perspective, (B) mesoscopic perspective, and (C) microscopic perspective.

From a multiscale perspective, metacomposites demonstrate a unique ability to decouple and integrate various loss mechanisms and impedance matching properties. This decoupling mitigates the traditional interdependence, reduces the temperature dependence of EMWA performances, and provides robust support for excellent EMWA performances over a wide temperature range. Firstly, based on the principles of effective medium theory and impedance matching, the exceptional anti-reflection characteristics of metacomposites enable more EMWs to penetrate the interior, where energy is subsequently dissipated through multiple collision (Fig. 6A). The discretized architecture of functional units effectively suppresses the percolation threshold for macroscopic conductive network, generating localized charge accumulation regions. This structural design achieves unprecedented decoupling between impedance matching characteristic and loss capability at elevated temperatures. Secondly, the sophisticated architecture facilitates multiscale interactions between incident EMWs and functional units, where mesoscopic-level multiple reflections and scattering phenomena prolong the propagation path of EMWs (Fig. 6B). This markedly increases the interaction sites between EMWs and units, further increasing the opportunities for EMWs to interact with matter, meaning that more EMWs are attenuated until they are exhausted, which is beneficial for the strong loss ability [20,55]. Thirdly, the EM dissipation characteristics are fundamentally governed by the intrinsic properties of microscopic components, which engender synergistic loss mechanisms, such as the strong polarization loss and enhanced conductive loss (Fig. 6C) [56–61]. Specifically, the 1D TiN NFs and 2D RGO architectures provide the abundant reflection channels and high-density polarization sites (including heterogeneous interfaces and functional group) that amplify interfacial and dipole polarization [48,59,62]. Furthermore, the 3D conductive networks formed through intercomponent connectivity facilitate efficient charge carrier migration, thereby substantially enhancing conductive loss [63–68]. Concurrently, the accumulation and uneven distribution of charges at heterogeneous interfaces generate macroscopic electric moments due to the difference in dielectric properties, thereby promoting EM energy dissipation [54]. Leveraging the synergy and complementarity of these multiscale effects, highly efficient EMWA is achieved across a wide temperature range. Overall, the architectural strategy of functionally discrete units with compositional and structural modulation not only optimizes impedance matching through spatially controlled dispersion but also elucidates the fundamental relationship between emergent loss mechanisms and EMWA performance, which ultimately generates exceptional EMWA performance with the varied temperatures.

## Conclusion

In summary, the TiN/RGO units with tailored conductivities and sizes are fabricated via an electrostatic spraying technique and subsequently assemble to construct the metacomposite. The discrete distribution of these units effectively suppresses the formation of continuous macroscopic conductive networks, thereby contributing to excellent impedance matching and reduced temperature sensitivity in EMWA performance. Notably, the subwavelength-scale units create a surface with exceptional microwave-trapping characteristics, facilitating the penetration of incident EMWs into the interior of the metacomposite. This results in additional energy dissipation through repeated collisions involving multiple reflections and scattering events. These processes extend the propagation path of EMWs within metacomposite, enhancing energy–matter interactions and increasing overall attenuation. Furthermore, the precise tailoring of the microscopic composition strengthens both conductive and polarization losses, which contribute to the superior EMWA performance. Considering these loss mechanisms, metacomposites exhibit remarkable EMWA performances (C2/PDMS metacomposites: −44.1 dB; S1/PDMS metacomposites: −48.1 dB) with an EAB covering almost the entire X-band at only 1 wt% across a wide temperature range (298 to 573 K). Thus, these findings provide valuable insights into multiscale loss mechanisms in metacomposites, optimizing composition and structure to enhance energy dissipation pathways and establishing a solid foundation for rational design strategies aimed at high-performance EMWA metacomposites under variable temperature conditions.

## Materials and Methods

### Materials

Tetrabutyl titanate [Ti(OC_4_H_9_)_4_] was purchased from Tianjin Komeo Chemical Reagent Co. Polyvinylpyrrolidone and zirconium acetate [Zr(OAc)_4_] were purchased from Aladdin Chemical Reagent Co. GO was prepared by the Hummers’method based on our previous work [27]. Polydimethylsiloxane (PDMS, 184 Silicone Elastomer) was purchased from Dow Corning Corporation. Anhydrous ethanol was purchased from Anhui Ante Biochemical Co.

#### Preparation of TiN/RGO units with different conductivities

TiO_2_ NFs were prepared by electrostatic spinning according to our previous work [13]. Subsequently, the homogeneous mixed solutions were prepared by blending the as-spun TiO₂ NFs with GO dispersions at varying mass ratios (1:0, 1:0.5, 1:1, and 1:1.2). The mixing process involved initial homogenization using a high-speed shear emulsifier for 5 min, followed by further agitation via magnetic stirring for 2 h to ensure uniform dispersion. The resulting mixture was then subjected to electrostatic spraying under controlled parameters: a positive voltage of 8.8 kV and a negative voltage of 0 kV with a precise injection rate of 1.2 mm/min to obtain microspheres. These microspheres were subsequently transferred to a freeze-dryer and maintained at −67 °C for 48 h to thoroughly remove water content via sublimation, resulting in the dry units with a controlled size of approximately 1.0 mm. Then, the units were heated at 5 °C/min to 900 °C under NH3 atmosphere and kept for 4 h for nitride reduction treatment, thereby converting TiO₂ into TiN and reducing GO to RGO. Through this process, a series of functional units with systematically tailored electrical conductivities were successfully obtained, which were labeled accordingly as C0, C1, C2, and C3, respectively.

#### Preparation of TiN/RGO units with different scales

Following the same steps described in the previous section, the preparation of units with different sizes was carried out under consistent conditions except for 2 key variables: the mass ratio of TiO₂ NFs to GO dispersion was fixed at 1:1, and the voltage parameters of the electrostatic spraying process were systematically adjusted. Specifically, the positive high voltage was varied to 16.5, 8.8, 6.2, and 2.4 kV, thereby obtaining 0.5-, 1-, 2-, and 3-mm units, which were marked as S0, S1, S2, and S3, respectively; S1 is the same as C2.

#### Preparation of TiN/RGO/PDMS metacomposites

The as-prepared functional units were blended with PDMS as the matrix and poured into cuboid molds (22.86 mm × 10.16 mm × 2 mm) and degassed, and then arranged manually. Following this, the samples were transferred to an oven and cured at 80 °C for 3 h to achieve complete cross-linking of the PDMS matrix. After demolding, the TiN/RGO/PDMS metacomposites were obtained with a low filler content of 1.0 wt% functional units. It mainly suggested that the excessive filler loading would increase the probability of contact between units, leading to the formation of macroscopic conductive pathways, causing impedance mismatching and reducing the ability of EMWs to penetrate into the metacomposites. Therefore, a low filling content (1.0 wt%) could achieve better EMWA performances.

### Characterization

The structure and composition of units were analyzed and characterized by an SEM (Carl Zeiss Gemini 500), an x-ray diffractometer (D8-Advance), and a Raman spectrometer (Hariba FRANCE SAS, laser excitation wavelength: 532 nm; exposure time: 3 s). The conductivity of unit was measured by using a dual electric 4-probe tester (Guangzhou Four-Probe Technology Company, RTS-9).

### EM measurement

The EM parameters of metacomposites were measured using a vector network analyzer (Ceyear, 3672B-S) across the X-band frequency range (8.2 to 12.4 GHz). During high-temperature measurements, a controlled heating procedure was applied, and the temperature was increased at a rate of 5 °C/min under a continuous argon atmosphere. Each target temperature point was stabilized and held for 3 min to ensure thermal equilibrium and measurement reliability. The reflection loss curves of metacomposites were calculated based on the transmission line theory. The EM energy loss capacity can be intuitively demonstrated by electric field vector distributions using computer simulation technology microwave studio (10 GHz) [24]. Models I, II, III and IV were constructed, corresponding to Cx (C0, C1, C2, and C3)/PDMS metacomposites. Models a, b, c, and d represent Sx (S0, S1, S2, and S3)/PDMS metacomposites, among which Model b is consistent with Model III.

## Data Availability

All data are available in the main text or the Supplementary Materials. Source data are available from the corresponding authors upon reasonable request.
