# Supplementary material for: Enhanced Microwave Trapping and Loss Capabilities of TiN/RGO/PDMS Metacomposites across a Wide Range of Temperatures
Source: Research (Wash D C). 2025 Oct 29;8:0972. doi: 10.34133/research.0972 (PMC12569479; doi:10.34133/research.0972)
Supplement: Supplementary 1 — Figs. S1 to S7 [file research.0972.f1.zip › Revised Supplementary material.docx]

**Enhanced Microwave Trapping and Loss Capabilities of TiN/RGO/PDMS Metacomposites Across a Wide Range of Temperatures**

Haoxu Si^1,2#^, Shuai Zhang^1#^, Yaqin Ding^1^, Chongyang Chai^1^, Shuaishuai Zhou^3^, Cuiping Li^1*^, Chunhong Gong^2*^, Jingwei Zhang^1*^

*1.* *National & Local Joint Engineering Research Center for Applied Technology of Hybrid Nanomaterials, Henan University, Kaifeng 475004, China*

*2. Institute of Functional Polymer Composites,* *College of Chemistry and Molecular Sciences, Henan University, Kaifeng 475004, China*

*3. School of Energy Science and Technology, Henan University, Kaifeng 475004, China*

*Corresponding author. E-mail: 10370020[@henu.edu.cn](mailto:@henu.edu.cn) (C. Li); gong@henu.edu.cn (C. Gong); jwzhang@henu.edu.cn (J. Zhang)

^#^These authors contributed equally to this work.

**Figure S1** SEM images of C0

**Figure S2** Real part (a) and imaginary part (b) of Cx/PDMS metacomposites at 298K

**Figure S3** The tangent value (a-d) of Cx/PDMS metacomposites at 298-573 K:

(a) C0, (b) C1, (c) C2 and (d) C3

**Figure S4** (a) S0, (b) S1, (c) S2 and (d) S3 units


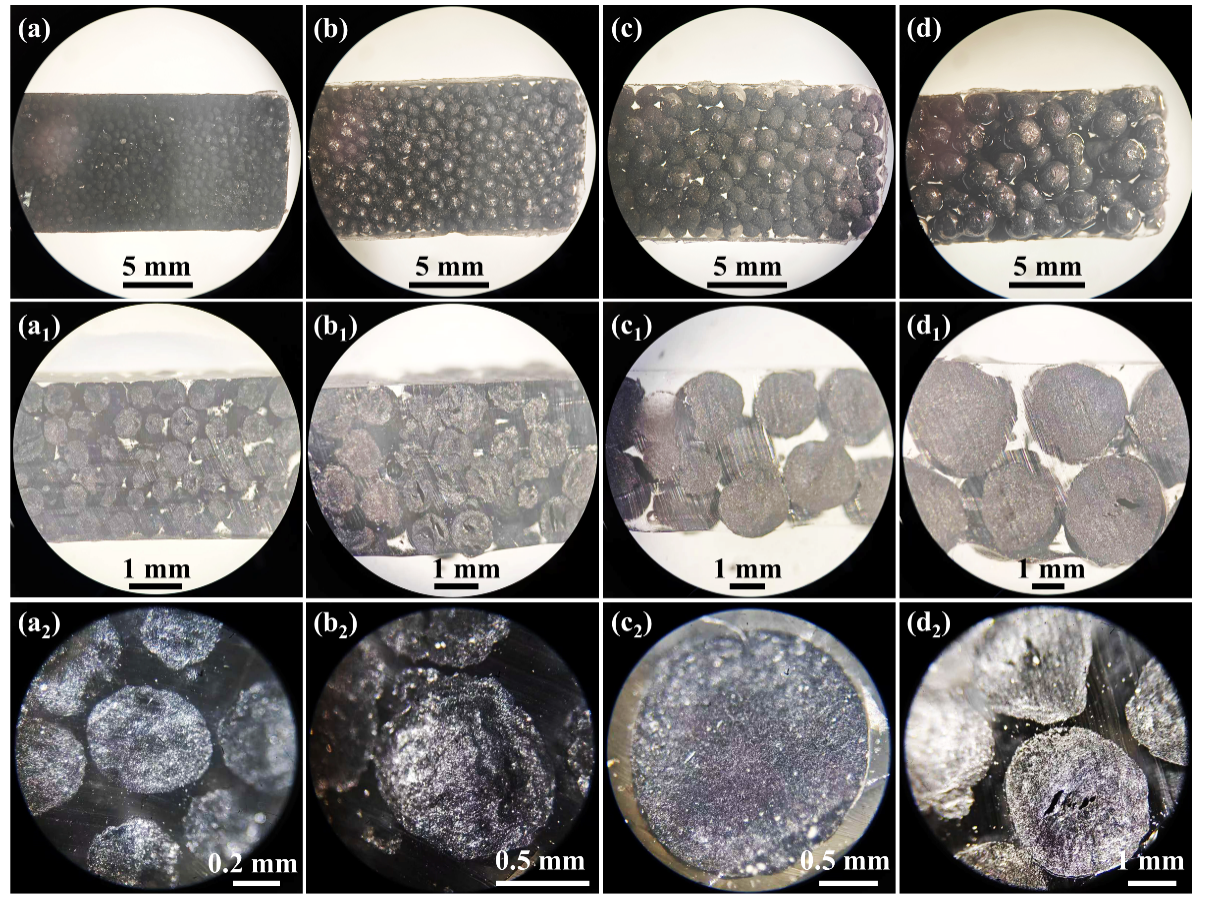


**Figure S5** The electronic images of Sx/PDMS metacomposites captured at various magnifications: (a-a_2_) S0, (b-b_2_) S1, (c-c_2_) S2 and (d-d_3_) S3

**Figure S6** Real part (a) and imaginary part (b) of Sx/PDMS metacomposites at 298K

**Figure S7** The tangent value (a-d) of Sx/PDMS metacomposites at 298-573 K:

(a) S0, (b) S1, (c) S2 and (d) S3
