# Supplementary figures and images for: Enhanced Microwave Trapping and Loss Capabilities of TiN/RGO/PDMS Metacomposites across a Wide Range of Temperatures
Source: Research (Wash D C). 2025 Oct 29;8:0972. doi: 10.34133/research.0972 (PMC12569479; doi:10.34133/research.0972)

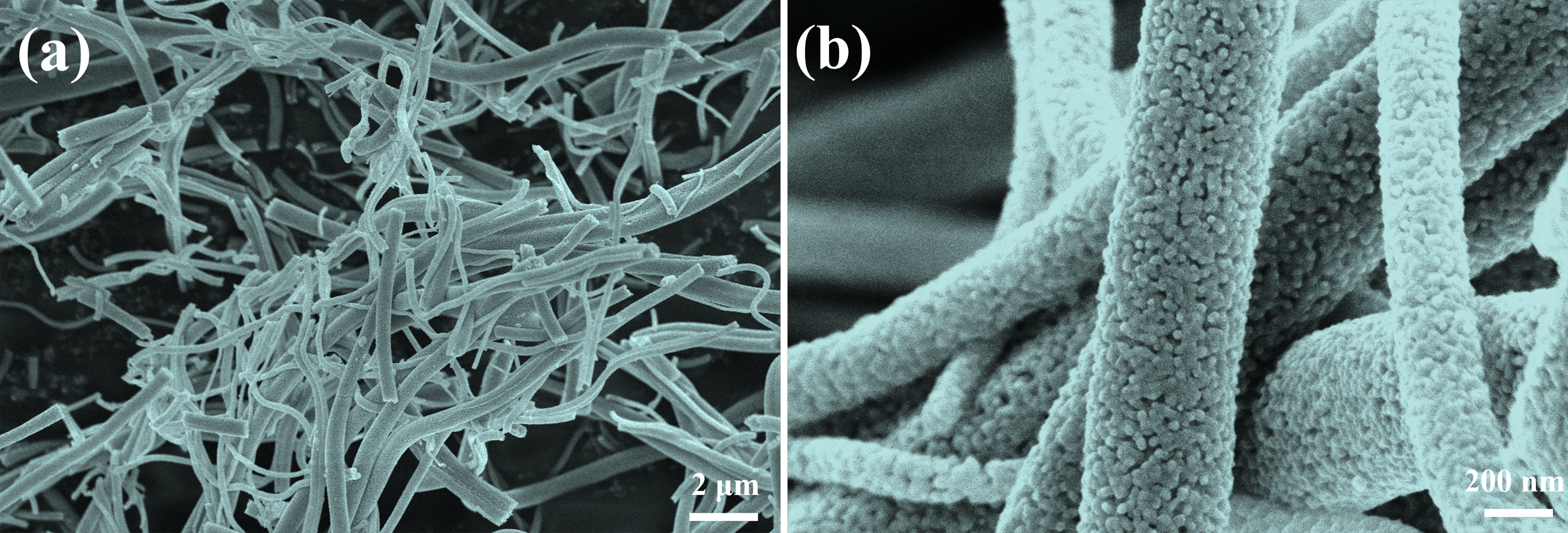

Supplement: Supplementary 1 — Figs. S1 to S7 [file research.0972.f1.zip › Figure S1.tif]

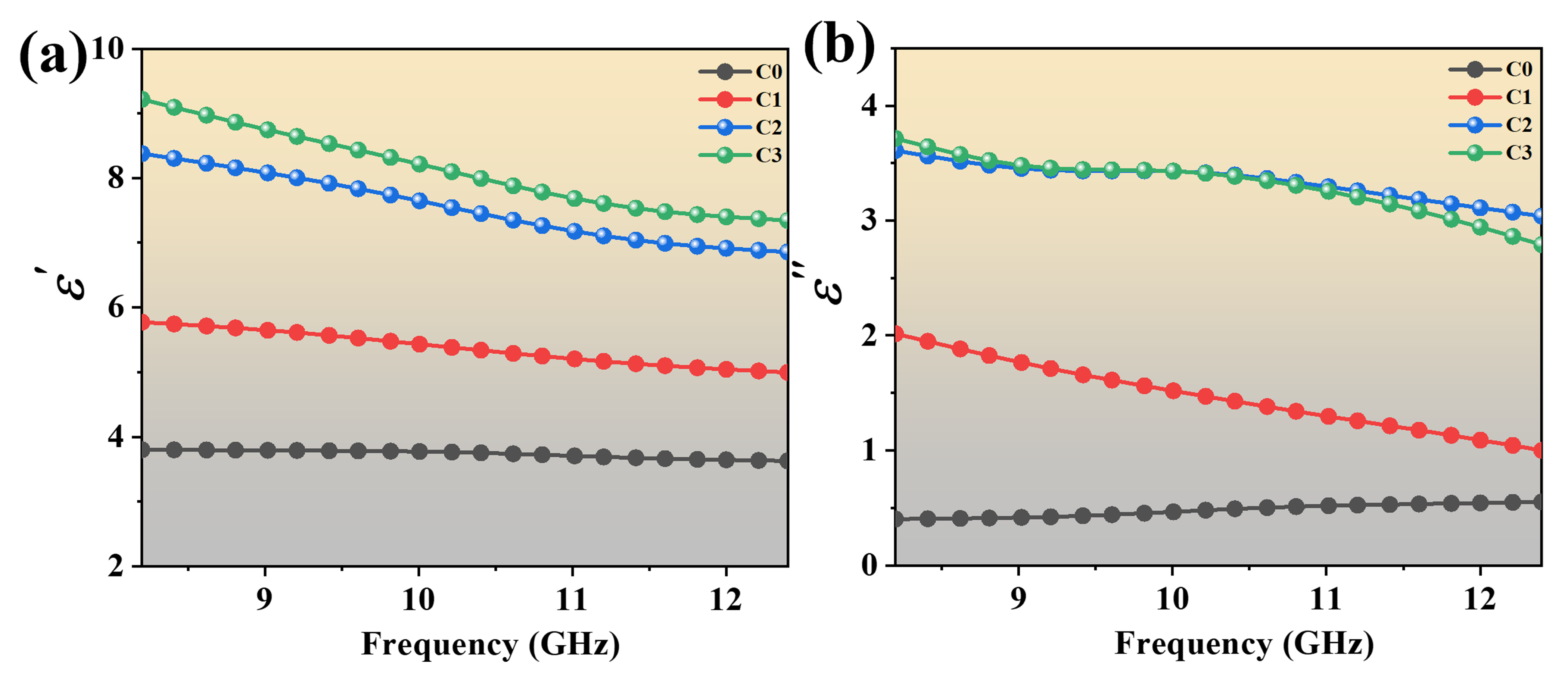

Supplement: Supplementary 1 — Figs. S1 to S7 [file research.0972.f1.zip › Figure S2.tif]

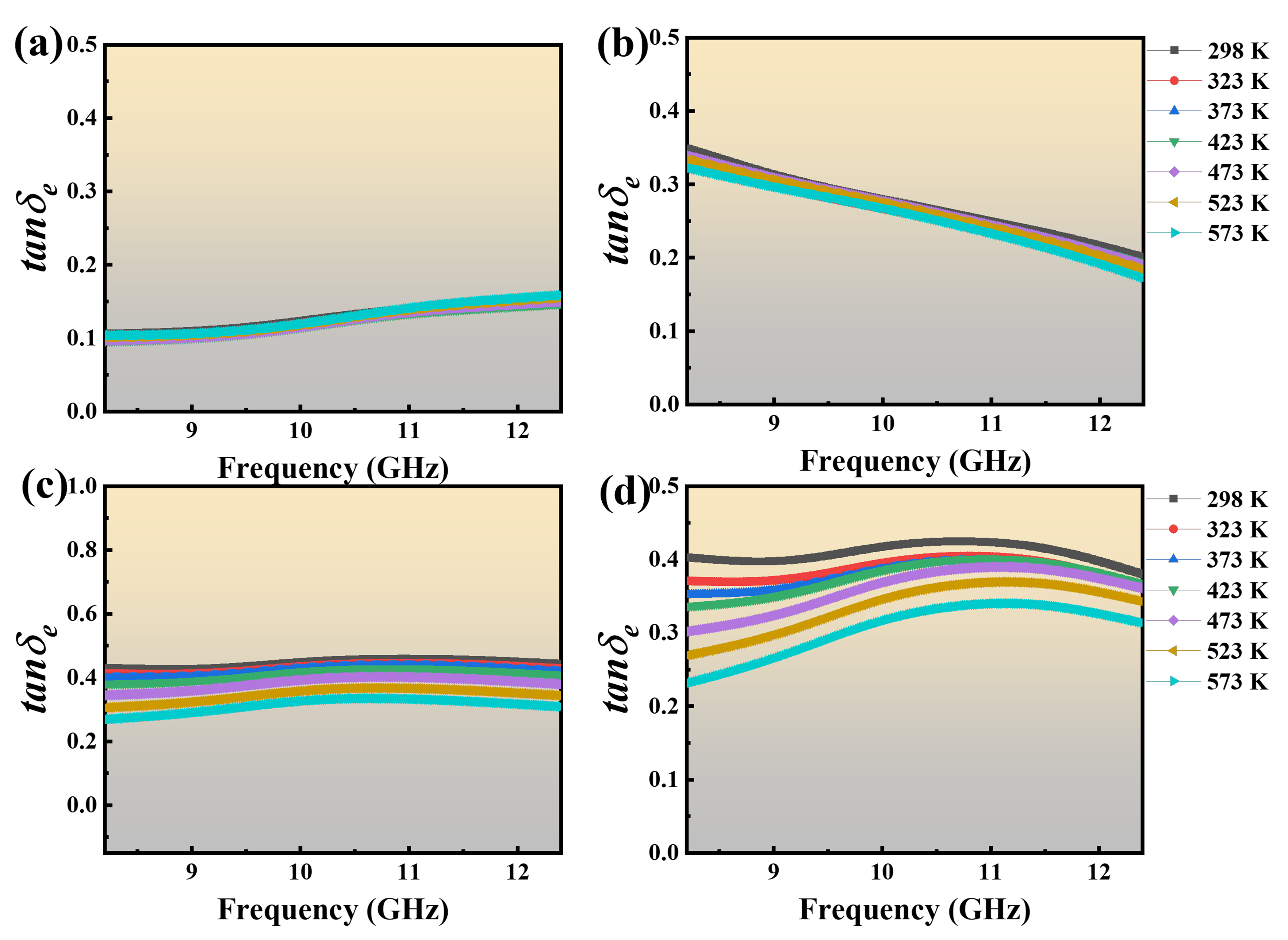

Supplement: Supplementary 1 — Figs. S1 to S7 [file research.0972.f1.zip › Figure S3.tif]

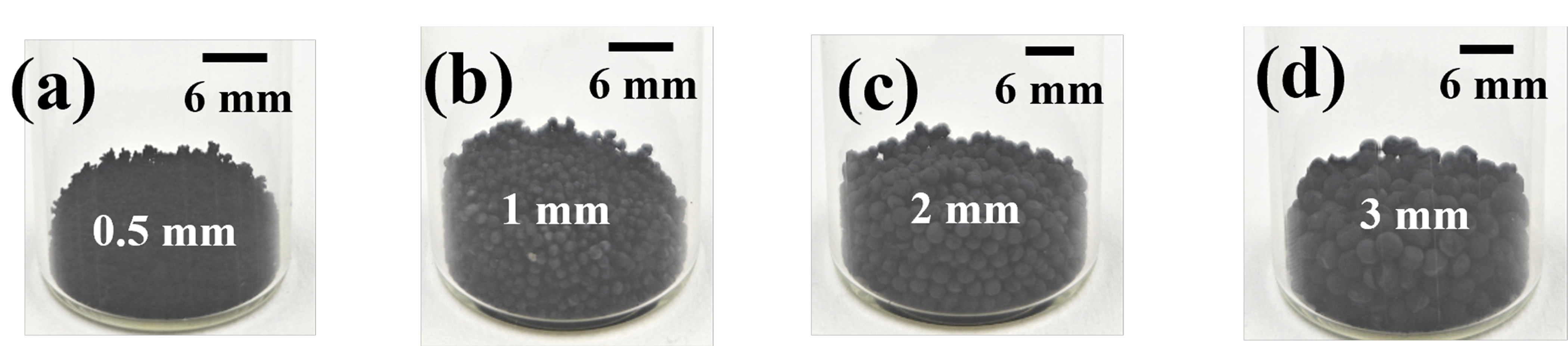

Supplement: Supplementary 1 — Figs. S1 to S7 [file research.0972.f1.zip › Figure S4.tif]

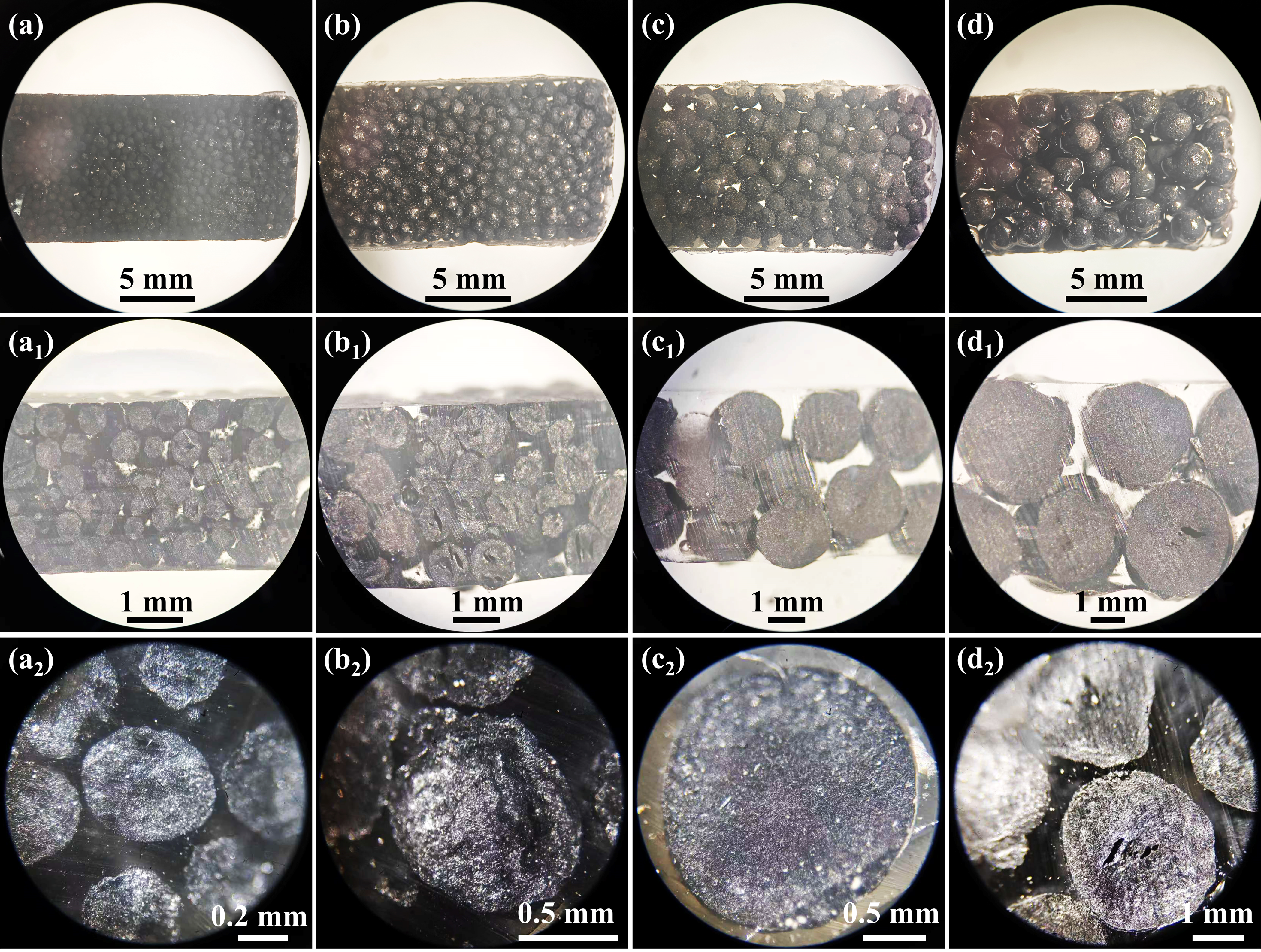

Supplement: Supplementary 1 — Figs. S1 to S7 [file research.0972.f1.zip › Figure S5.tif]

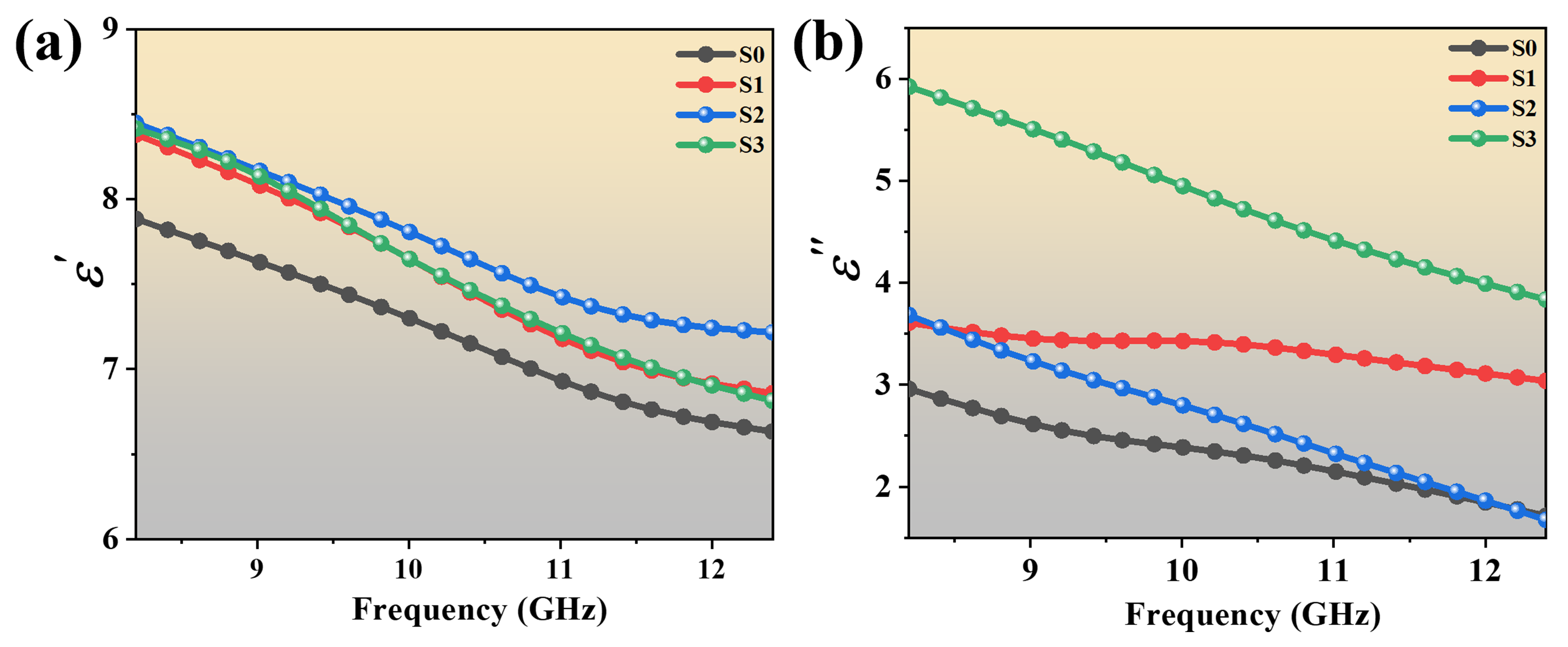

Supplement: Supplementary 1 — Figs. S1 to S7 [file research.0972.f1.zip › Figure S6.tif]

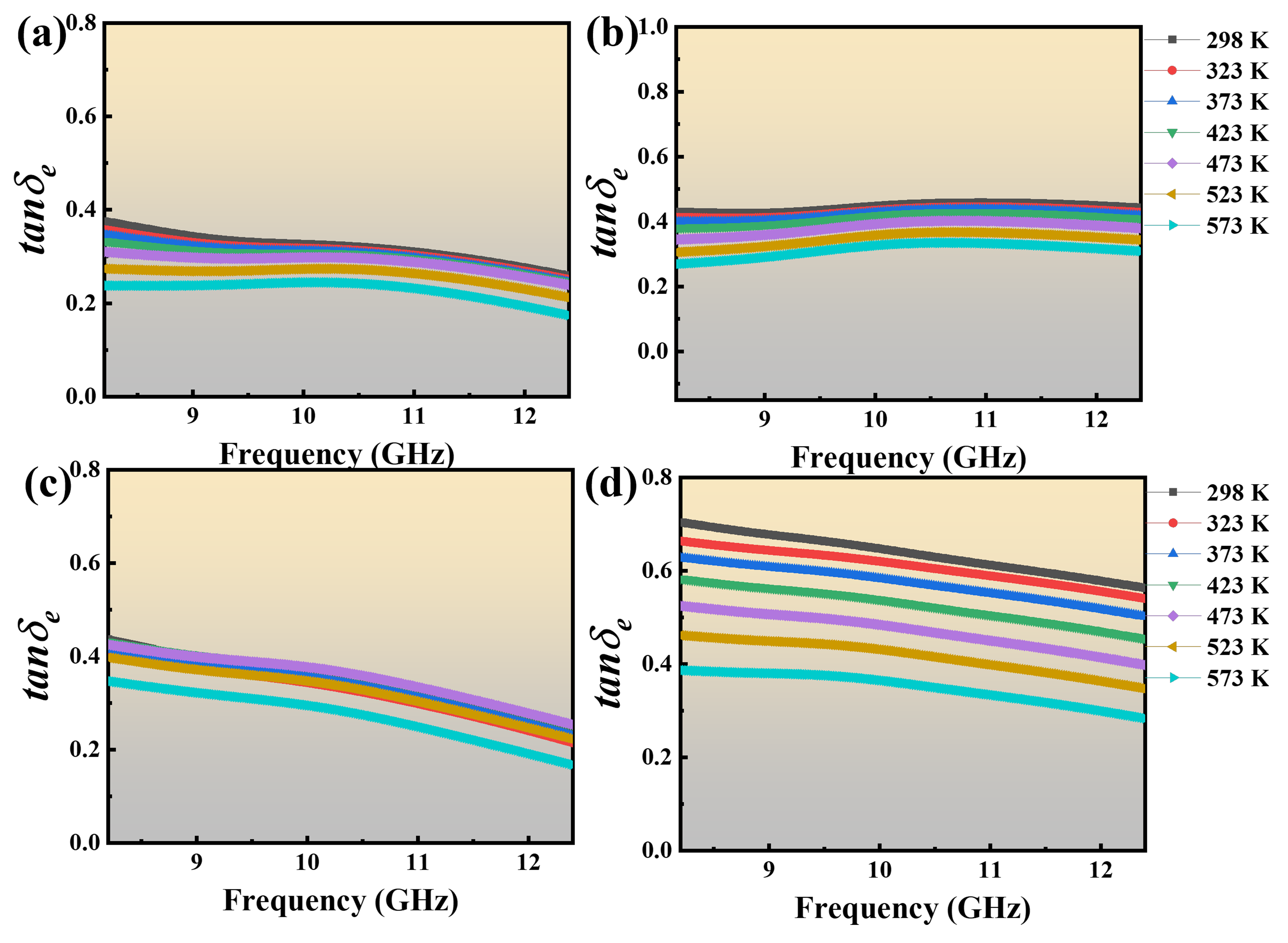

Supplement: Supplementary 1 — Figs. S1 to S7 [file research.0972.f1.zip › Figure S7.tif]
